# Language Development in Deaf and Hard-of-Hearing Children: An Ecological Systems Perspective

**DOI:** 10.3390/audiolres16040103

**Published:** 2026-07-23

**Authors:** Carrie A. Davenport, Elaine R. Smolen, Derek M. Houston

**Affiliations:** 1 Building Bridges Consulting LLC, Columbus, OH 43202, USA; 2 Department of Health Studies and Applied Educational Psychology, Teachers College, Columbia University, New York, NY 10027, USA; es3519@tc.columbia.edu; 3 Department of Speech, Language, and Hearing Sciences, University of Connecticut, Storrs, CT 06269, USA; derek.houston@uconn.edu

**Keywords:** language, deaf, ecological systems, cochlear implants, hearing aids

## Abstract

**Background**: Language outcomes among DHH children are widely variable. Equally variable is the diversity of factors involved in fostering healthy language development in this population. Some DHH children use cochlear implants and hearing aids and reach expected milestones. Others exhibit delays, ranging from mild to severe. Regardless of how “success” is defined for individual children who use these technologies, recognizing the ecological contexts in which DHH children develop language, and the relationships among those contexts, can support the identification of contextual predictors and points of intervention that lie beyond the individual DHH child. **Purpose**: The purpose of this article is to articulate Bronfenbrenner’s Ecological Systems Theory and how it relates to language development in this population of children. We discuss the original conceptualization of the theory, portraying the levels of ecosystems depicted as nested, and explore how a networked version of the theory might provide a complementary lens for elucidating the ecological variables associated with language development. **Implications for research:** We describe implications for research including expanding the ecological variables examined, examining interactions between ecosystems, use of diverse methodologies, and shifting beyond a deficit model towards an ecological perspective.

## 1. Introduction

DHH children develop language within multiple interacting ecological systems that extend beyond the parent–child dyad. Meinzen-Derr et al. [1] report the challenges this group of children face in developing language skills are often multifactorial (e.g., presence of intellectual or developmental disabilities, availability of appropriate educational settings) [2], and intersectional factors such as families’ access to healthcare and intervention services and economic stability [3]. The complexity of the challenges requires an interdisciplinary approach to generating new knowledge, expanding our understanding of “what works,” and developing and testing evidence-based interventions [4], all in service of promoting better outcomes for more DHH children. Addressing complex scientific topics by leveraging cross-disciplinary expertise of scientists and community-based participatory research methods [5] has gained traction in recent years [6]. By integrating expertise across disciplines related to language development in DHH children (e.g., family science, education, speech and hearing sciences, and linguistics) and the lived experiences of associated communities (i.e., DHH adults, families), scientists can enhance the rigor and relevance of their research.

**The purpose of this article is to contextualize the ecosystems in which DHH children develop language.** We begin with an overview of Ecological Systems Theory (EST) [7], a framework commonly used to motivate child development research [8]. EST asserts the influence of environments, and the interconnectedness of those environments, on child development [7]. The theory has been particularly valuable in identifying contextual predictors or points of intervention that lie beyond personal characteristics in children with disabilities, such as the role of specialist teachers for children with visual impairments [9] and inclusive classroom practices for preschoolers [10]. Here we describe the original conceptualization of the theory, depicted as a series of nested ecosystems with the developing child at the center, each level reflecting environments both proximal (e.g., family) and distal (e.g., society) directly and indirectly influencing development.

Building upon Bronfenbrenner’s original conceptualization of the theory, we consider the view of the theory in which the ecosystems are networked rather than nested [11]. Based on social network theory [12], Neal and Neal argue the nested model implies proximal ecosystems as the most potent variables related to development as compared to distal ecosystems. Rather, they view the relationships among ecosystems as overlapping, conferring a more complex set of ecological variables directly and indirectly related to development. Here, we discuss this alternate conceptualization of EST not as a substitute but complementary to the original, for exploring developmental pathways for DHH children. We conclude with implications for research, including expanding the ecological variables examined, examining interactions between ecosystems, use of diverse methodologies, and shifting beyond a deficit model towards an ecological perspective.

## 2. Ecological Systems Theory of Child Development

Bronfenbrenner argued that, rather than studying human development over time in an isolated manner, human development should be studied “in the actual environments in which human beings lived their lives” [13]. To more fully interpret human behavior and enhance our understanding of it, scientists must examine aspects of children’s environments, not only on a micro- or macro-level but how different ecosystems interact and ultimately, impact development [7]. The focus of most studies motivated by EST has been related to the influence of *context*, or setting-level influences, on development [11].

The research model associated with EST, referred to as PPCT, consists of the following four primary components: process, person, context, and time [13]. *Processes* are the interactions between the developing child and other people, objects and/or symbols in their immediate environment. To influence development, processes—parent–child interaction and joint engagement are two examples—must occur frequently over time and grow progressively complex [13]. *Person* refers to the individual of interest or to specific characteristics (e.g., DHH child or parental hearing status) and can be treated as an antecedent of proximal processes or an outcome of those processes [13]. Examinations of joint engagement as it relates to a particular hearing level [14] or parental hearing status and child proficiency in English and ASL [15] are examples of treating personal characteristics as an antecedent variable. Level of speech perception as a function of age of cochlear implantation is an example of a personal characteristic treated as an outcome of the habilitation process [16].

In the PPCT model, *context* serves as a critical variable in relation to development. Context receives the most attention from Bronfenbrenner, as he was concerned that scientists were not sufficiently considering the ecosystems in which children engage. A variety of variables can serve as *context* in examining proximal processes. Nevertheless, the selection of contextual variables must be theoretically driven and relevant to the other variables under examination [17]. A prime example is socio-economic status (SES), a contextual variable that can be measured and evaluated in relation to processes associated with language development. Spanning from [18] study of parental language use as a function of parental educational level to more recent research examining joint engagement in children from low-income households [19], context is a critical feature of developmental research. SES has also been a key contextual variable in language development research with DHH children [20]; however, other contextual variables are also important (e.g., family functioning, household dynamics [21]) and prime for further investigation.

*Time* is considered on multiple levels in the EST model. Microtime is the continuity versus discontinuity of ongoing episodes of proximal process and reflects the extent to which participant(s) are focused upon the episode [13]. An example with DHH children is the evidence indicating hearing parents engage in fewer episodes of joint attention [22] and joint engagement with their children compared to hearing parents with hearing children [23]. Mesotime relates to the frequency with which the developing individual engages in a proximal process, over the course of days, weeks, and years. Proximal process requires both micro- and mesotime, that is, the interaction must occur relatively regularly over an extended period [13]. This is an area where researchers can build upon current work, examining the longitudinal effects of proximal processes (e.g., parent–child interaction) and later language outcomes in DHH children. Macrotime refers to pertinent historical events and evolving social norms across the process of individual development as well as generations. Here, we might consider the expansion of cochlear implant (CI) candidacy criteria (i.e., a change that has happened over time) or the inclusion of Deaf adults who use American Sign Language in popular television shows and movies and how that might impact parents’ perceptions of sign use with their DHH children.

### 2.1. Systems as Nested

**Figure 1:**
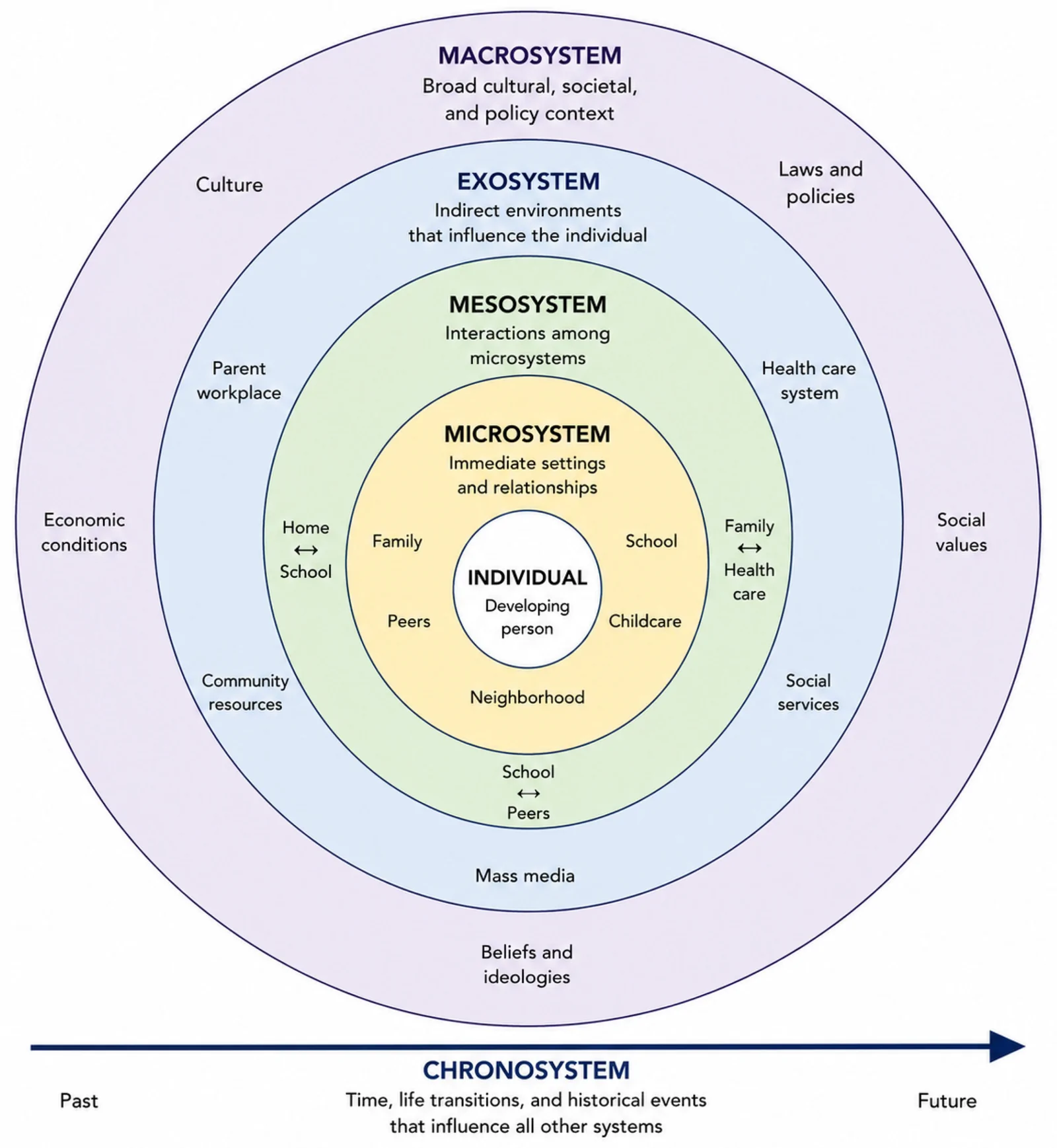
Bronfenbrenner’s Ecological Systems Theory as nested. **Note.** Concentric circles depict nested ecological systems surrounding the developing individual. The chronosystem represents the influence of time and life transitions across all ecological levels. Original illustration created by the authors based on Bronfenbrenner [7], Bronfenbrenner [36] and Bronfenbrenner and Morris [14].

Bronfenbrenner’s initial articulation of EST described the levels of context, processes and time, or ecosystems surrounding the individual of focus, as “nested” within one another. See Figure 1 for a depiction of the nested model.

According to this model, the microsystems are the immediate environments in which children play a direct role and have the most potent influence on development [7]. Thus, unsurprisingly, the parent–child dyad has been and continues to be a primary focus of studies related to language development in children, with and without disabilities [19,24,25]. Parent interactions have long been a critical focal point of researchers interested in language development in DHH children [26,27,28]. The literature on social interactions at school among DHH children and hearing peers has also received substantial attention [29,30,31], particularly as increasing numbers of DHH children are educated in general education settings rather than self-contained DHH classrooms [32]. However, there is a dearth of research related to DHH children’s experience outside of the school setting [33]. A synthesis of qualitative studies that gathered the perspectives of *hearing in everyday life* (i.e., listening in traffic, contending with classroom noise) among DHH children and youth, challenging listening conditions and strategies to manage the experience of hearing, were identified as major themes. Critical to this discussion is the need for more direct input from DHH children about challenges and supportive strategies to better support inclusive practices in educational and community settings [33].

Expanding outwards are the mesosystems, the relationships and interactions between major microsystems, such as school and home or home and community. The integration between these most proximal systems can either promote or inhibit children’s development [13]. For DHH children, these ecological systems include the multiplicity of microsystems they experience (i.e., home, school, community, and in the case of children who use hearing technology, the clinical setting) and the interactions between those ecosystems. Communication and collaboration among families, audiology and CI clinics and educational settings is challenging but important for families navigating these settings [34]. Families function as the hub of services because communication between clinicians and school-based providers is typically limited due to large caseloads and competing schedules [35]. The challenges of the relationship between clinics and schools have been acknowledged [34] and there are examples of efforts to improve communication and seek ways to collaborate so that children with technology can maximize their potential.

An extension of the mesosystem, the exosystem consists of formal and informal societal structures that may not directly influence the developing child, but nonetheless influences the developmental process (e.g., mass media, governmental agencies). In the nested model, this outer level of influence on development might be a large clinical caseload at the facility where DHH children receive therapy, or community resources that pertain to DHH children; each of these exosystem variables have the potential to indirectly impact language development. The DHH child may not interact directly with these exosystems, but they affect the child’s life [36].

The macrosystem is the overarching pattern of the micro-, meso-, and exosystems attributed to a particular culture. The broader cultural, societal, and economic factors that influence the child’s environment include national policies, social norms and ideologies. The outermost ring is the chronosystem, the change or consistency over time related to individual and environmental characteristics. The chronosystem refers to the dimension of time, that is, the changes and events that happen over the course of a child’s life (i.e., health crisis) or family transitions (e.g., moving, divorce). Macrosystems and chronosystems are not necessarily derived from settings; therefore, the first three systems of the theory are of primary interest [11].

### 2.2. Systems as Networked

Some social scientists [12] argue the nested arrangement implies that participation in the smallest circle means participation in the larger systems. McPherson [37] theorized that the nested model of ecosystems is anachronistic to contemporary society. In this view, the social structures in earlier societies were relatively limited, in which social networks consisted of fewer individuals, experiences, and environments [37]. Neal and Neal [11] assert two primary advantages to adopting the networked model, both of which we believe have value in terms of advancing language development research with DHH children. The first advantage cited is the emphasis on the extent to which development is a social process involving interactions between ecosystems. The second is the potential for examining more complex relationships among ecological systems.

The networked model of EST focuses less on *where* one interacts and more on *how* and *with whom* one interacts. Much effort has been made to explain differences in language outcomes in DHH children centered on language learning context, the *where* in terms of language program (i.e., listening and spoken language, total communication, ASL–English bilingual) [38]. For example, Dettman et al. [38] examined speech perception and expressive vocabulary among children implanted before age 4 years and compared outcomes among children enrolled in different “intervention programs” described as auditory verbal, auditory oral, and bilingual–bicultural. As reported, all children in each group used the same “communication approach” before and after implantation. Each approach is described in terms of the goals of the program (e.g., in auditory–oral programs children are encouraged to use listening and visual cues to develop spoken language).

This approach has generated knowledge critical to understanding some contextual factors associated with stronger language and skills. However, the relationship between language input and language outcomes is complex for children with hearing technology [39]. Technology must be in working order; clinicians must be trained in the unique challenges DHH children face in meeting language milestones; school settings must be selected based on appropriate assessments, input from Individual Education Program teams, individual needs of the child, and oftentimes requires in-service training for teachers unfamiliar with educating DHH children. Therefore, what appears as “distal” factors potentially have a direct or indirect influence on the trajectory of a DHH child’s development. Large clinical caseloads, long waiting periods for appointments, and a shortage of qualified teachers of the deaf has created a multi-faceted challenge for many families advocating for their DHH child.

The second advantage of including a networked lens along with a nested approach is that it motivates researchers to examine more complex relationships among ecological systems [11]. For DHH children, these ecosystems include the multiplicity of microsystems they experience (i.e., home, school, community, and clinic) and the interactions between those systems. Communication and collaboration among families, audiology and CI clinics and educational settings is challenging [34]. Families function as the hub of services because communication between clinicians and school-based providers is often limited due to large caseloads and competing schedules [34,35].

Some clinicians and educators seek to mitigate the stress on families as they recognize families oftentimes function as the “go-between” among home life, school, and clinical care [40,41]. Some major CI centers employ staff dedicated to liaising with the educational audiologists, speech-language pathologists, and teachers who work with their patients [42]. Outreach efforts at special schools for DHH children and centers supported by state education agencies (e.g., Ohio Center for Autism and Low Incidence) also seek to bridge the divide between the ecosystems [43]; however, major knowledge gaps exist related to the efficacy of these programs.

Multi- and inter-systems research is needed to examine the effects of these efforts and testing methods for improving communication and collaboration between families, clinics and schools. Further research is needed related to the perspectives of providers on the educational experiences of DHH children with CI [44,45]. Involving families in the research process is another avenue for identifying contextual predictors or points of intervention that lie beyond child characteristics [46].

### 2.3. A Networked Ecological Framework for Language Development in DHH Children

We view the nested and networked perspectives of EST not as competing frameworks but complementary for understanding the complex ecological influences on language development in DHH children. The nested perspective is a helpful structure for organizing ecological variables according to proximity to the developing child. The networked perspective emphasizes the dynamic interactions among the individuals, entities and organizations, and systems that shape DHH children’s language development. Together, these perspectives encourage researchers to shift beyond identifying isolated predictors of language outcomes and toward examining how interconnected ecological systems generate opportunities for—or barriers to—language development.

**Figure 2:**
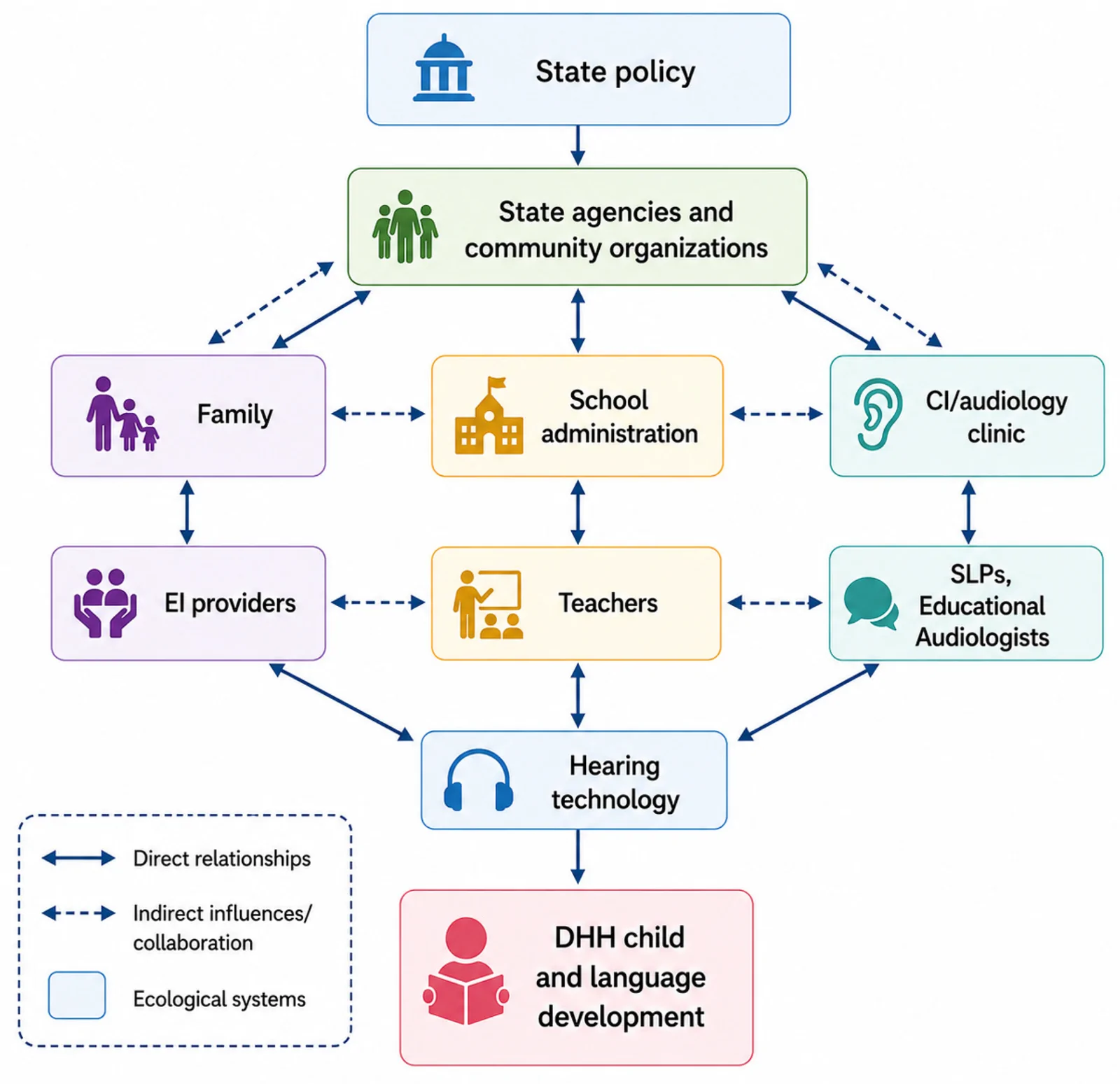
Proposed conceptual network integrating nested and networked ecological perspective for language development in DHH children.

Figure 2 presents a conceptual framework illustrating the interconnected nature of these ecological systems as they relate to language development in DHH children. Instead of presenting ecological systems as separate hierarchical layers, this framework emphasizes the connections between families, clinicians, EI providers, educational settings, community organizations and policy makers. Children’s access to language, EI, education, and family support is influenced by the strength and quality of these relationships. Viewing these ecological systems as interconnected highlights the potential for generating research questions that examine individual ecological variables and the relationships within them.

## 3. Implications for Language Research with DHH Children

Adopting a nested and networked view of EST not only provides alternate avenues for organizing ecological variables, but these perspectives also offer a framework for identifying malleable influences on language development. Moreover, they offer a framework for generating interdisciplinary research questions and informing interventions that extend beyond the parent–child dyad. Viewing the networked perspective as complementary rather than replacing the traditional perspective broadens the scope of inquiry by emphasizing the interactions among ecological systems that collectively shape language experiences in DHH children. We offer the following implications for adopting a nested and networked perceptive of EST.

**Implication** **1.** *Expand the selection of ecological variables examined.*

The first implication is the need for expanding the selection of ecological variables in relation to DHH children and language development. Much of current research focuses on parent responsiveness [26], family environment [47,48] and SES [49]. Future work should also examine educational placement and communication among providers based on previous research [34,50,51] indicating real challenges for children and families in these areas. Future research should also focus not only on access to hearing technology, but access to Deaf adults and community resources, intervention and services that support bilingual or multimodal language development and factors associated with cultural–linguistic identity development. Current studies demonstrate disparities to some services based on multiple family- and system-level factors [3,52]. Policy is another ecological variable in need of further research so that we can understand the influences of various policy efforts, such as LEAD-K [53] and Madeline’s Law [54], on language outcomes.

**Implication** **2.** *Investigate interactions between systems.*

The second implication is the need for investigating interactions between systems. Four major research questions exist. First, under what ecological conditions does family environment predict language development? Second, how do strong collaborations between schools and CI clinics, or schools and outreach organizations influence language outcomes? Third, does family engagement moderate the effects of educational placement? Fourth, what is the relationship between provider and family communication and parental self-efficacy?

**Implication** **3.** *Use novel methodologies.*

A third implication is the use of novel methodologies, such as social network analysis, community-based participatory research (CBPR) and mixed methods. First, there is potential for social network analysis to identify the individuals and entities (e.g., family-based organizations) most influential in fostering language development in DHH children. Second, CBPR could identify ecological barriers overlooked by researchers. Last, quantitative and qualitative mixed methods could explain why quantitative associations between ecological variables and language outcomes occur.

**Implication** **4.** *Moving beyond child deficits.*

**Figure 3:**
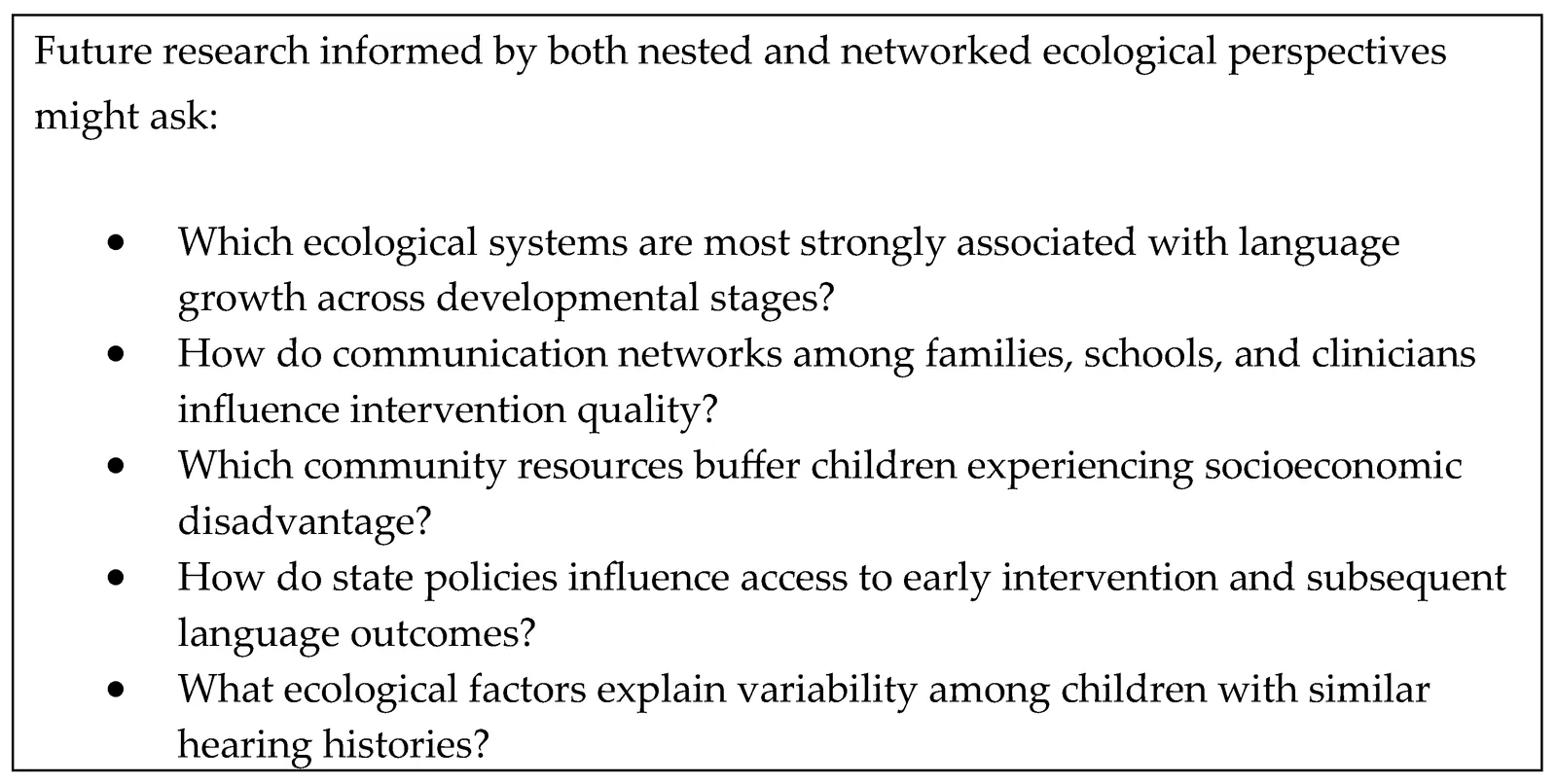
Potential research questions.

The fourth implication is the shift in focus when considering language development in DHH children—moving from studying why individual DHH children demonstrate poorer language outcomes to examining ways in which systems generate or reduce opportunities for language development. See Figure 3 for potential research questions.

## 4. Future Directions

Ultimately, adopting both nested and networked ecological perspectives encourages researchers to shift beyond isolated predictors toward understanding how interacting ecosystems shape language development in DHH children. This approach has the potential to generate interventions that are child-centered and system-centered and address barriers within families, schools, healthcare systems, and communities. Viewing ecological systems as interconnected networks emphasizes the importance of building bridges across ecological systems, among families, educators, clinicians and community stakeholders rather than studying each system separately.

## Data Availability

No new data were created or analyzed in this study. Data sharing is not applicable to this article.
